# Germplasm Screening and Transcriptome Profiling Identify Phenylpropanoid Biosynthesis-Related PAL Candidate Genes Associated with Freezing Tolerance in Potato

**DOI:** 10.3390/biom16091249

**Published:** 2026-08-28

**Authors:** Yuwei Ge, Qianqian Wang, Yuying Fu, Tingting Wang, Huajun Liao, Chongchong Yan

**Affiliations:** 1 Institute of Vegetables, Anhui Academy of Agricultural Sciences, Hefei 230001, China; 2 The Key Laboratory of Horticultural Crop Germplasm Creation and Utilization (Jointly Built by MARA and Anhui Province), Hefei 230001, China; 3 The Key Laboratory of Horticultural Crop Germplasm Creation and Efficient Cultivation of Anhui Province, Hefei 230001, China

**Keywords:** potato (*Solanum tuberosum* L.), freezing tolerance, germplasm accessions, transcriptome, phenylpropanoid biosynthesis, candidate genes

## Abstract

Potato (*Solanum tuberosum* L.) seedlings are highly sensitive to freezing temperatures, which severely impairs growth and causes substantial losses in tuber yield and quality. This study aimed to screen freezing-tolerant potato germplasm accessions, identify candidate genes involved in the freezing response, and provide elite parental materials and a theoretical reference for molecular breeding of freezing-tolerant cultivars. We evaluated seedling freezing tolerance of 73 potato accessions using the freezing damage index (FDI), and performed transcriptome sequencing on leaf samples from highly freezing-tolerant (HT) accession 15-1881 and highly freezing-susceptible (HS) accession B8 following freezing treatment. Substantial variation in freezing tolerance was observed across the germplasm panel, with 5 HT accessions and 12 HS accessions identified, showing marked phenotypic and physiological differences under freezing stress. Transcriptomic analysis detected 6560 differentially expressed genes (DEGs) in 15-1881 and 5161 DEGs in B8, with 3558 DEGs specific to 15-1881. The phenylpropanoid biosynthesis pathway exhibited noticeable expression divergence between 15-1881 and B8, harboring 17 15-1881-specific DEGs including three tandem phenylalanine ammonia-lyase (PAL) genes. The HT germplasm accessions obtained in this study provide breeding resources for freezing-tolerant potato improvement, and the *PAL* genes characterized here represent promising candidate genes associated with freezing response in potato.

## 1. Introduction

Potato is a staple food and vegetable crop widely cultivated throughout China [1,2]. Freezing injury at the seedling stage represents a major abiotic constraint limiting potato production [3,4]. Although potato is categorized as a cool-season crop, its seedlings have poor freezing tolerance [5]. Freezing temperatures disrupt cellular homeostasis and can cause seedling mortality and substantial tuber yield loss [6]. Therefore, developing freezing-tolerant cultivars and elucidating the underlying molecular regulatory mechanisms are core strategies for mitigating frost-related losses.

Considerable progress has been made in deciphering potato freezing tolerance mechanisms. Previous studies have revealed that osmotic adjustment, reactive oxygen species scavenging, and cell membrane stability constitute key physiological adaptive responses under freezing stress [7,8]. Several freezing-responsive transcription factors and functional genes have been identified via transcriptomics and molecular experiments, most of which were obtained from limited cultivated materials or single genetic backgrounds [9].

Currently, two mainstream systems are used to evaluate potato freezing tolerance: field phenotyping under natural frost conditions and indoor artificial freezing treatment [10]. Field identification relies on variable natural temperature conditions and exhibits poor repeatability [11]. Conventional laboratory assessment, represented by measurement of the semi-lethal temperature (LT_50_), is labor-intensive and unsuitable for primary screening of large germplasm populations [12,13]. In contrast, the freezing damage index (FDI) evaluates frost injury based on visual phenotypic grading of seedlings, allowing rapid phenotypic recording. Indoor freezing stress treatment combined with calculation of the FDI enables rapid, reproducible primary phenotypic assessment of dozens of germplasm accessions; this method was therefore adopted as our core evaluation approach in this study.

RNA sequencing (RNA-seq) has been widely applied to identify stress resistance-related candidate genes in horticultural crops [14,15,16,17,18]. The phenylpropanoid biosynthesis pathway plays critical roles in reactive oxygen species (ROS) scavenging and cell membrane stabilization in various Solanaceae species under abiotic stress [19,20,21]. However, the regulatory role of this pathway in potato freezing responses remains unclear. Furthermore, few studies have integrated germplasm phenotypic screening with comparative transcriptomics to explore the molecular mechanisms of freezing tolerance in potato. Against this backdrop, critical knowledge gaps persist in germplasm exploration and the linkage between phenotypic freezing responses and underlying molecular events.

In this study, we adopted an integrated strategy combining germplasm screening and comparative transcriptome profiling. We carried out freezing-tolerance phenotyping on 73 potato germplasm accessions using the FDI assay to identify contrasting highly freezing-tolerant (HT) and highly freezing-susceptible (HS) accessions. We next compared morphological and physiological responses of these contrasting accessions under standardized freezing stress to characterize phenotypic differences associated with divergent freezing performance. Finally, we performed comparative transcriptome sequencing using one representative HT accession (15-1881) and one representative HS accession (B8) selected from the germplasm panel to screen candidate genes potentially associated with potato freezing response. This study provides germplasm and candidate genes for potato freezing tolerance breeding.

## 2. Materials and Methods

### 2.1. Experimental Materials

A total of 73 potato germplasm accessions were provided by the Institute of Vegetables, Anhui Academy of Agricultural Sciences, comprising 31 landraces, 23 released cultivars, and 19 hybrid progeny lines (Table S1). All accessions were maintained in the germplasm resource bank of the same institute.

### 2.2. Method for Freezing Tolerance Evaluation of Potato

#### 2.2.1. Seedling Cultivation

In vitro plantlets of each accession were subcultured on fresh MS medium and incubated at 22 °C under 2000 l× with a 14 h light/10 h dark photoperiod. After three weeks of subculture, uniform and healthy seedlings were selected for hydroponic cultivation. For each potato accession, six seedlings were planted in one hydroponic box as one independent biological replicate, and three independent biological replicates were established for subsequent freezing treatment. All hydroponic boxes were randomly arranged in the growth chamber to minimize positional and container effects. The cultivar Favorita was used as a stable freezing-susceptible reference control, which had been widely validated as a freezing-sensitive potato cultivar.

Hydroponic seedlings were cultured with Hoagland’s nutrient solution, which was refreshed every three days. Seedlings were cultivated at 22 °C under 10,000 l× with a 14 h light/10 h dark photoperiod. Freezing tolerance evaluation was performed after 2 weeks of hydroponic acclimation.

#### 2.2.2. Freezing Tolerance Assay

All hydroponic seedlings were transferred to a low-temperature chamber at −4 °C for 3 h, followed by a 7-day recovery period under normal growth conditions. After recovery, the freezing injury grade of each seedling was recorded visually, and the freezing damage index (FDI) was calculated accordingly. Five freezing injury grades (1, 3, 5, 7, 9) were established based on seedling freezing symptoms (Table S2). The FDI was calculated via Formula (1):

(1) $$FDI=\sum \frac{N_{i}\times {FD}_{i}}{N_{t}\times {FD}_{h}}\times 100\%$$

where FDI = freezing damage index; N_i_ = number of seedlings with grade-i freezing injury; FD_i_ = score corresponding to the i-th freezing injury grade; N_t_ = total number of seedlings evaluated; FD_h_ = maximum injury grade score.

All germplasm accessions were classified into five freezing tolerance groups based on FDI values: highly freezing-tolerant (HT, 0.00 < FDI ≤ 0.20), freezing-tolerant (T, 0.20 < FDI ≤ 0.40), moderately freezing-tolerant (MT, 0.40 < FDI ≤ 0.60), susceptible (S, 0.60 < FDI ≤ 0.80), highly susceptible (HS, 0.80 < FDI ≤ 1.00) (Table S3).

### 2.3. 3,3′-Diaminobenzidine (DAB) Histochemical Staining

The youngest fully expanded leaves of potato seedlings were collected for DAB staining to detect in situ hydrogen peroxide accumulation. Staining procedures were performed according to the manufacturer’s instructions of the Plant Tissue ROS Detection Kit (DAB method, Cat. No. G4815; Solarbio, Beijing, China). After staining, leaf samples were boiled in ethanol until all green pigmentation faded. Stained leaves were photographed, with brown precipitates indicating sites of hydrogen peroxide accumulation in leaf tissues.

### 2.4. Measurement of Physiological Indices

Malondialdehyde (MDA), soluble sugar, and proline contents were determined using corresponding commercial assay kits from Solarbio Science & Technology Co., Ltd., Beijing, China (MDA: Cat. No. BC6410; soluble sugar: Cat. No. BC0035; proline: Cat. No. BC0290). Fresh aboveground tissues were sampled for MDA and proline measurement, while oven-dried and pulverized aboveground tissues were applied for soluble sugar quantification.

The youngest fully expanded leaves were collected to determine electrolyte leakage rate and chlorophyll content, according to previously reported methods with minor modifications [22,23]. For electrolyte leakage measurement, fresh leaf tissues were rinsed with deionized water and immersed in ultrapure water at room temperature. The electrical conductivity of the ultrapure water blank (EC_0_) was measured first. After 30 min of incubation, the initial conductivity of the solution containing leaf samples (EC_1_) was determined. Subsequently, the samples were boiled for 30 min to release total electrolytes, and then cooled to room temperature before measuring the total conductivity (EC_2_). The relative electrolyte leakage rate was calculated as (EC_1_ − EC_0_)/(EC_2_ − EC_0_) × 100%. Leaf chlorophyll content was quantified via spectrophotometric detection after ethanol extraction.

### 2.5. Transcriptome Sequencing and Data Analysis

The freezing-tolerant line 15-1881 and the freezing-susceptible line B8 were selected for comparative transcriptome sequencing. Seedlings under normal growth conditions were set as the control group, while seedlings exposed to freezing stress at −1 °C for 4 h were defined as the stress group. The top three young leaves from each seedling were sampled, with three biological replicates per group. RNA libraries were sequenced on the BGI T7 platform. Raw sequencing reads were filtered to obtain high-quality clean reads, which were aligned to the potato reference genome DM v6.1. Gene expression abundance was quantified as FPKM values following data normalization. DEGs were identified using the thresholds of adjusted *p*-value (*padj*) < 0.05 and |log2(fold change)| ≥ 1. GO and KEGG enrichment analyses were performed using the R package clusterProfiler (v4.12.0). Gene sets from the potato reference genome DM v6.1 were used as the background gene universe. Up- and downregulated DEGs were combined for joint enrichment analysis. Significantly enriched terms were defined with *padj* < 0.05.

### 2.6. Quantitative Real-Time PCR (qRT-PCR)

Total RNA was extracted from potato leaf tissues using the RNAprep Pure Plant Kit (TIANGEN BIOTECH (BEIJING) Co., Ltd., Beijing, China). First-strand cDNA was reverse-transcribed from 2 μg of total RNA using PrimeScript II Reverse Transcriptase (Takara Biotechnology (Dalian) Co., Ltd., Dalian, China). qRT-PCR amplification was performed on a Bio-Rad real-time PCR instrument using the SYBR Premix Ex Taq™ Kit (TaKaRa) according to the manufacturer’s instructions. The potato elongation factor gene *StEF1α* was selected as the internal reference gene.

## 3. Results

### 3.1. Phenotypic Evaluation of Freezing Tolerance Indicators

Plant stress tolerance is commonly assessed using multiple growth-related phenotypic parameters, including survival rate (SR), biomass ratio (BMR), and seedling height ratio (SHR) [24,25,26,27,28]. In this study, we employed the FDI to quantitatively evaluate freezing tolerance in potato seedlings, which enables rapid phenotypic screening under controlled artificial freezing treatments in the laboratory.

**Figure 1:**
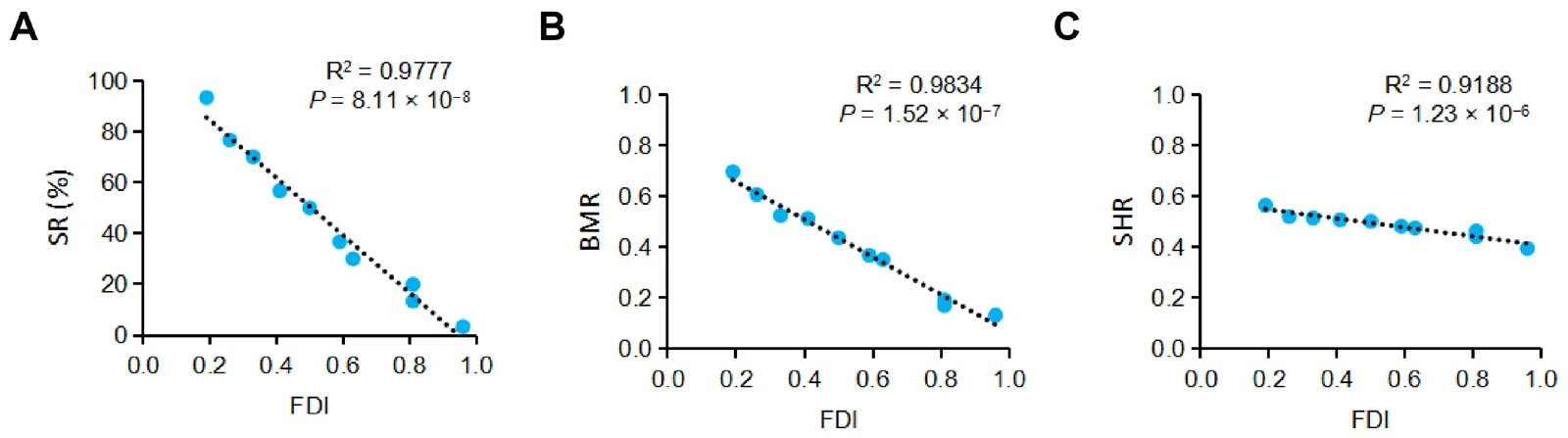
**Correlations between phenotypic indicators and freezing damage index (FDI).** (**A**) Correlation analysis between survival rate (SR, seedling survival rate under freezing stress) and FDI. (**B**) Correlation analysis between biomass ratio (BMR, the ratio of biomass under freezing stress to that of the control group) and FDI. (**C**) Correlation analysis between seedling height ratio (SHR, the ratio of seedling height under freezing stress to that of the control group) and FDI. Pearson correlation coefficients were calculated, and significance was assessed using a two-tailed *t*-test (*n* = 10).

To validate the reliability of FDI-based evaluation, we randomly selected 10 representative germplasm accessions covering the full gradient of freezing tolerance, including highly tolerant, moderately tolerant, and susceptible genotypes, to ensure representative phenotypic variation to simultaneously measure SR, BMR, SHR, and FDI, and performed correlation analysis. The results show that FDI exhibited strong negative correlations with SR (R^2^ = 0.9777, *p* = 8.11 × 10^−8^), BMR (R^2^ = 0.9834, *p* = 1.52 × 10^−7^), and SHR (R^2^ = 0.9188, *p* = 1.23 × 10^−6^), with all correlation coefficients significant at the 95% confidence level. These findings demonstrate that FDI can comprehensively reflect the overall freezing injury sustained by seedlings (Figure 1A–C). Compared with individual growth indicators, FDI reduces experimental labor input and is suitable for batch assessment of large germplasm populations; we therefore used FDI as the core evaluation index for subsequent population phenotypic characterization.

### 3.2. Freezing Tolerance Classification of 73 Potato Germplasm Accessions

**Figure 2:**
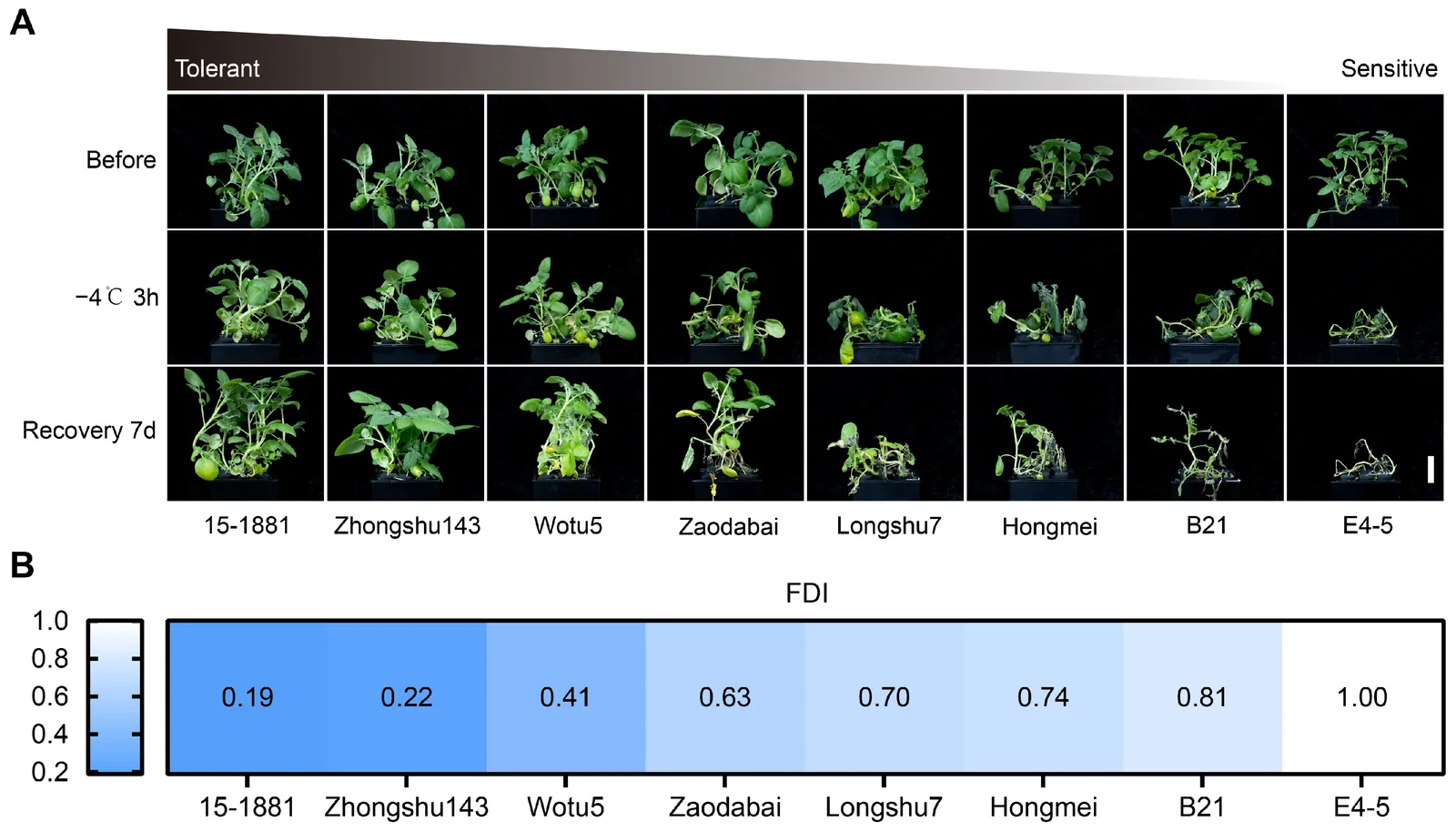
**Phenotypes of representative potato germplasm accessions under freezing stress.** (**A**) Phenotypic photographs of representative potato accessions before freezing treatment, after freezing treatment and recovery period, scale bar = 5 cm. (**B**) FDI values of the tested representative accessions. Data are presented as the mean of three biological replicates.

We evaluated the freezing tolerance of all 73 collected potato germplasm accessions. After −4 °C freezing treatment for 3 h followed by a 7-day recovery period, we observed significant inter-accession variation in freezing resistance (Figure 2A, Table 1). The reference control cultivar Favorita displayed typical freezing-sensitive phenotypes under this treatment with an FDI value of 0.81, which served as a stable phenotypic benchmark for assessing freezing damage severity across all germplasm accessions (Table 1). Representative HT accessions such as 15-1881 and 16HF001 (FDI = 0.19) showed nearly undamaged growth after recovery, whereas susceptible accessions E4-5 and B8 (FDI = 1.00) exhibited complete seedling mortality (Figure 2B, Table 1).

The freezing tolerance classification of all tested germplasm accessions is summarized in Table 1: 5 accessions (15-1881, 16HF001, E2-3, etc.) were classified as HT, 23 accessions (E1-3, G2, WJ2, etc.) were T, 18 accessions (1-2, 2019344036, Wotu5, etc.) were MT, 15 accessions (G4, G9, Zaodabai, etc.) were S, and the remaining 12 accessions (2-9, A1, B21, etc.) were HS. Highly freezing-tolerant materials accounted for only 6.85% of the total population (Figure S1), with only one HT accession identified among cultivars. By comparison, two hybrid progeny lines and two landraces showed greater breeding potential for the development of freezing-tolerant potato cultivars (Table 1, Figure S1).

### 3.3. Distinct Morphological and Physiological Responses Between Contrasting Freezing-Tolerant Germplasm Accessions

**Figure 3:**
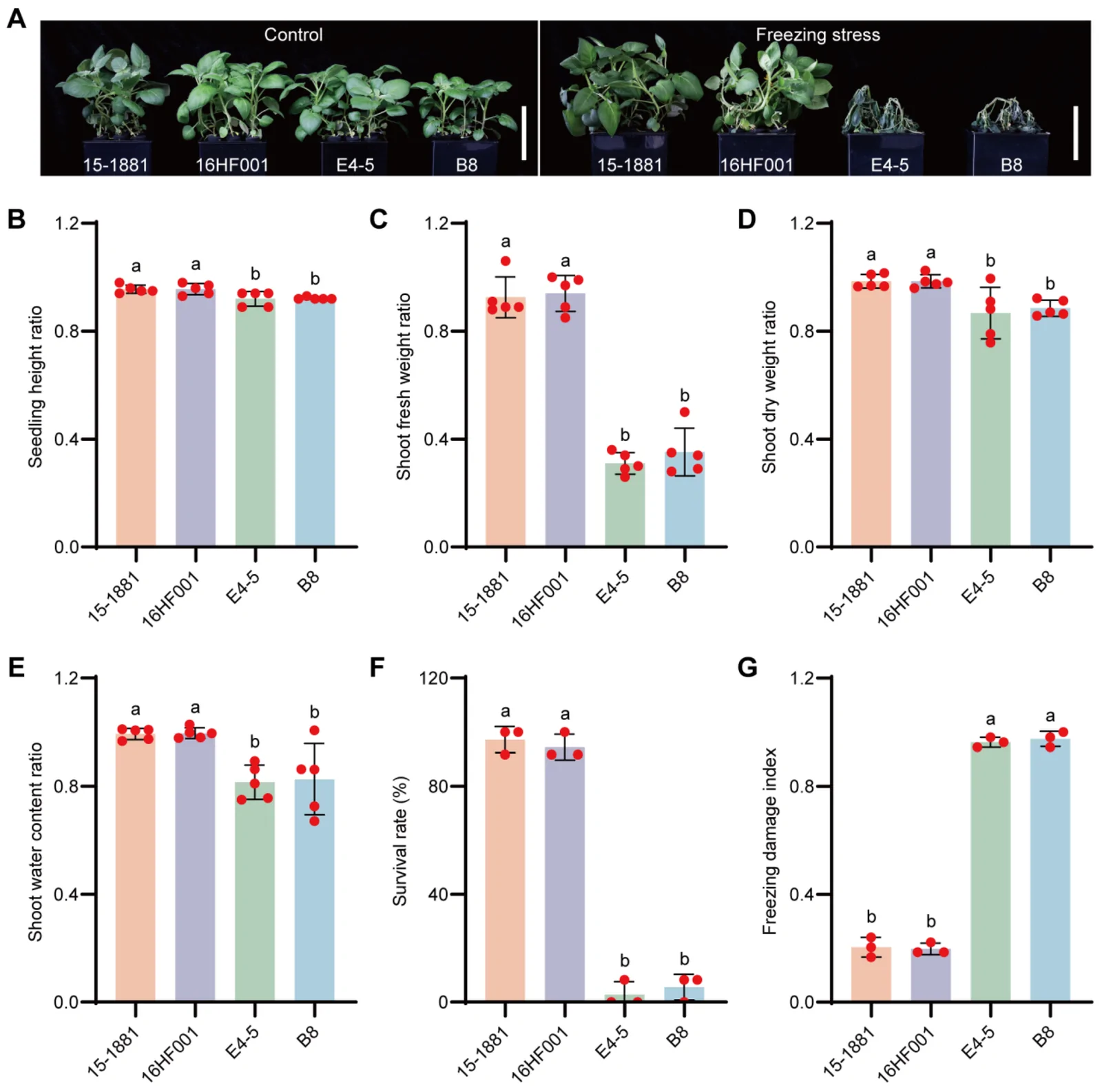
**Phenotypes of potato germplasm accessions with contrasting extreme freezing tolerance.** (**A**) Phenotypic photographs of accessions 15-1881, 16HF001, E4-5 and B8 under normal growth conditions (**left**) and freezing stress (**right**), scale bars = 10 cm. (**B**–**G**) Seedling height ratio (**B**), shoot fresh weight ratio (**C**), shoot dry weight ratio (**D**), shoot water content ratio (**E**), survival rate (**F**), and freezing damage index (**G**) of the four accessions. Data are presented as means ± SD, *n* = 5 for panels (**B**–**E**), *n* = 3 for panels (**F**,**G**). Different lowercase letters above bars denote significant differences between four accessions (*p* < 0.05, one-way ANOVA with Tukey’s HSD test).

To further compare phenotypic and physiological differences under freezing stress, we selected two HT accessions (15-1881, 16HF001) and two HS accessions (E4-5, B8) for systematic analysis (Figure 3A). Following −4 °C freezing treatment, HT accessions exhibited significantly higher seedling height ratio, shoot fresh weight ratio, shoot dry weight ratio, shoot water content ratio, and survival rate, along with markedly lower FDI values, compared with the HS accessions E4-5 and B8 (Figure 3B–G). These results indicate that HT accessions maintained stronger growth recovery capacity through higher biomass and survival rates and milder freezing damage.

**Figure 4:**
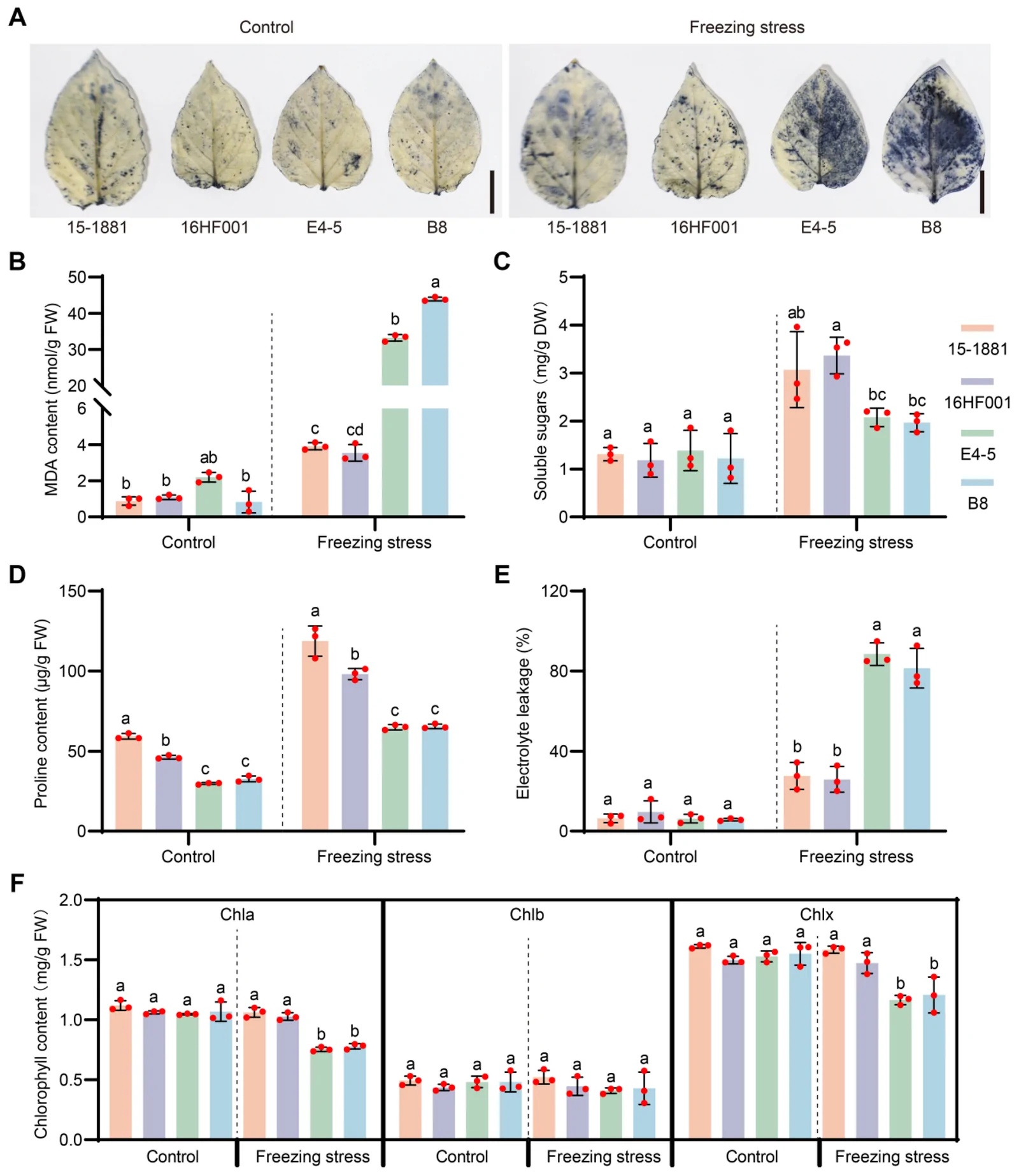
**Physiological responses of potato germplasm accessions with contrasting freezing tolerance.** (**A**) 3,3′-Diaminobenzidine (DAB) staining photographs of accessions 15-1881, 16HF001, E4-5 and B8 under normal growth conditions (**left**) and freezing stress (**right**), scale bars = 1 cm. (**B**–**E**) Malondialdehyde (MDA) (**B**), soluble sugar content (**C**), proline content (**D**), and electrolyte leakage rate (**E**) of the four accessions under normal growth conditions and freezing stress. (**F**) Contents of chlorophyll a (**left**), chlorophyll b (middle) and total chlorophyll (**right**) in the four accessions under normal growth conditions and freezing stress. Data from panels (**B**–**F**) are presented as means ± SD (*n* = 3). Different lowercase letters above bars denote significant differences among groups (*p* < 0.05, two-way ANOVA with Tukey’s HSD test).

Physiological measurements revealed clear biochemical differences between the two phenotypic groups. Freezing stress induces massive accumulation of ROS [29]. Excess ROS triggers membrane lipid peroxidation and substantial MDA production [4]. We performed DAB staining and MDA quantification to evaluate freezing-induced oxidative injury. The results show that HS accessions accumulated markedly higher levels of H_2_O_2_ and MDA in leaf tissues, suggesting greater oxidative damage, while HT accessions exhibited relatively lower oxidative stress levels (Figure 4A,B).

Freezing stress also promotes the biosynthesis of osmoprotectants (soluble sugar, proline) and disrupts cell membrane integrity, leading to increased electrolyte leakage [30]. We quantified soluble sugar content, proline content, and electrolyte leakage rate and found that HS accessions had significantly lower osmoprotectant concentrations and higher electrolyte leakage than HT materials under freezing stress (Figure 4C–E). HT germplasm accessions accumulate abundant soluble sugar and proline to maintain osmotic homeostasis and reduce membrane electrolyte loss.

We measured leaf chlorophyll contents and found that HS accessions exhibited significantly decreased chlorophyll a and total chlorophyll contents under freezing stress, while chlorophyll b levels remained relatively stable between tolerant and susceptible groups (Figure 4F). Collectively, germplasm accessions with differing freezing tolerance exhibited distinct morphological phenotypes and physiological homeostasis under freezing stress.

### 3.4. Transcriptome Profiling and Identification of Differentially Expressed Genes

**Figure 5:**
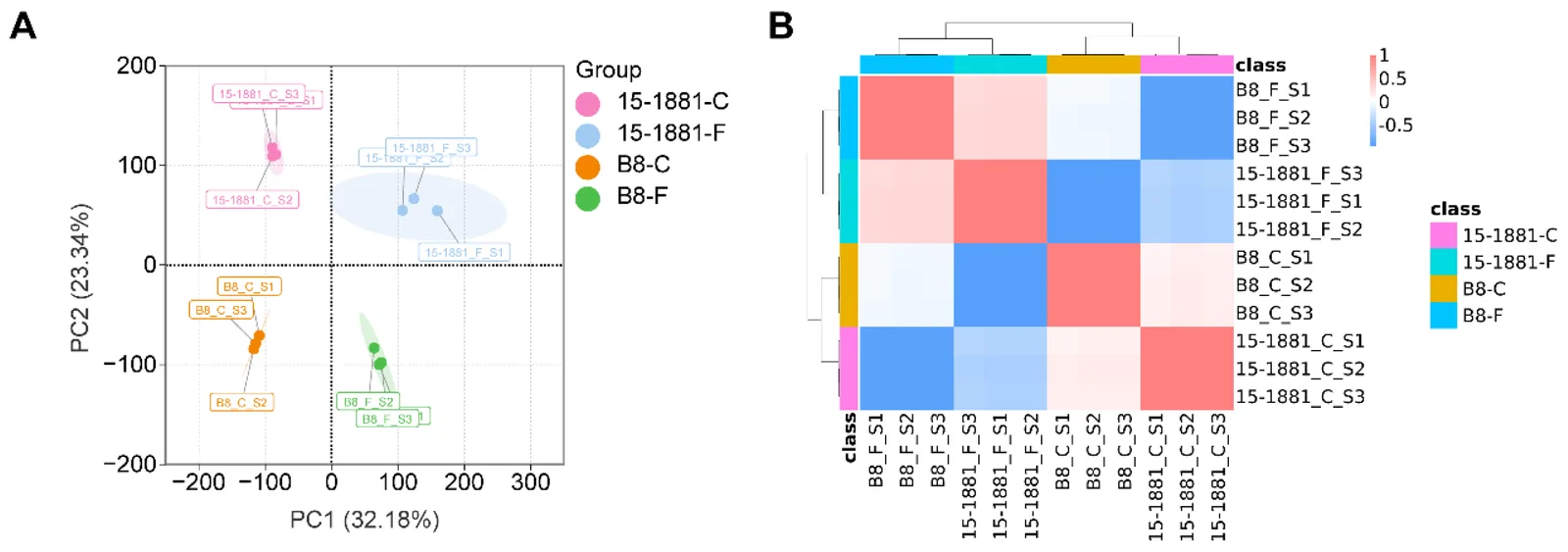
**Principal component analysis (PCA) and hierarchical clustering based on correlation of potato transcriptome samples.** (**A**) PCA revealed clear separation between control and freezing-stressed groups, with tight clustering of biological replicates indicating high reproducibility. (**B**) Sample correlation heatmap integrated with hierarchical clustering tree.

To elucidate the molecular regulatory differences between HT and HS phenotypes under freezing stress, we performed comparative RNA-seq on leaf samples from 15-1881 and B8. Four sample groups (15-1881 control, 15-1881 freezing treatment, B8 control, B8 freezing treatment) were set up, each with three biological replicates. All sequencing samples exhibited Q30 values above 94%, and GC content was within the typical range reported for potato (Table S4), confirming that the sequencing data were reliable for subsequent analyses. Principal component analysis (PCA) clearly separated samples by treatment and accession, with tight clustering of the three replicates within each group (Figure 5A). A Pearson correlation heatmap further verified high reproducibility and clear transcriptional divergence between the contrasting groups, with no outlier samples detected (Figure 5B).

**Figure 6:**
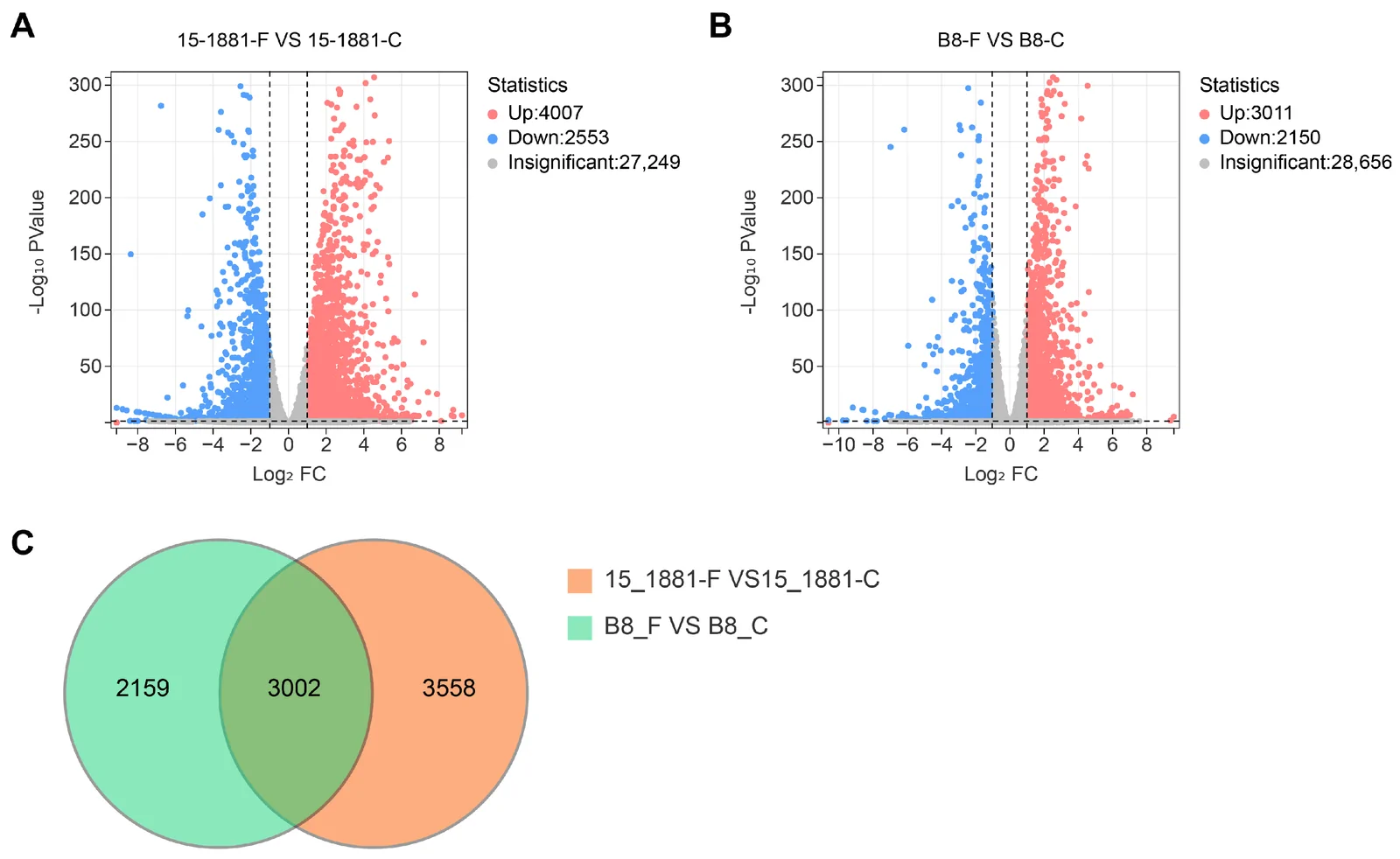
**Differentially expressed genes (DEGs) identified from potato transcriptome data.** (**A**) Volcano plot showing DEGs in the comparison between 15-1881-F and 15-1881-C. (**B**) Volcano plot showing DEGs in the comparison between B8-F and B8-C. (**C**) Venn diagram illustrating the shared and unique DEGs between the two comparison groups (15-1881-F vs. 15-1881-C and B8-F vs. B8-C). F = freezing stress; C = control.

We identified DEGs using the thresholds of |log_2_(fold change)| ≥ 1 and *padj* < 0.05. A total of 6560 DEGs (4007 upregulated, 2553 downregulated) were identified in 15-1881 after freezing treatment, while 5161 DEGs (3011 upregulated, 2150 downregulated) were detected in accession B8 under identical treatment conditions. Volcano plots indicate that the HT accession 15-1881 possessed a greater number of freezing-responsive DEGs than the HS accession B8 under the tested conditions (Figure 6A,B). Venn diagram analysis identified 3558 genotype-specific DEGs unique to 15-1881 and only 2159 unique DEGs in B8, with 3002 DEGs shared between the two accessions (Figure 6C).

### 3.5. Functional Enrichment and Expression Analysis of Freezing-Responsive Candidate Genes

**Figure 7:**
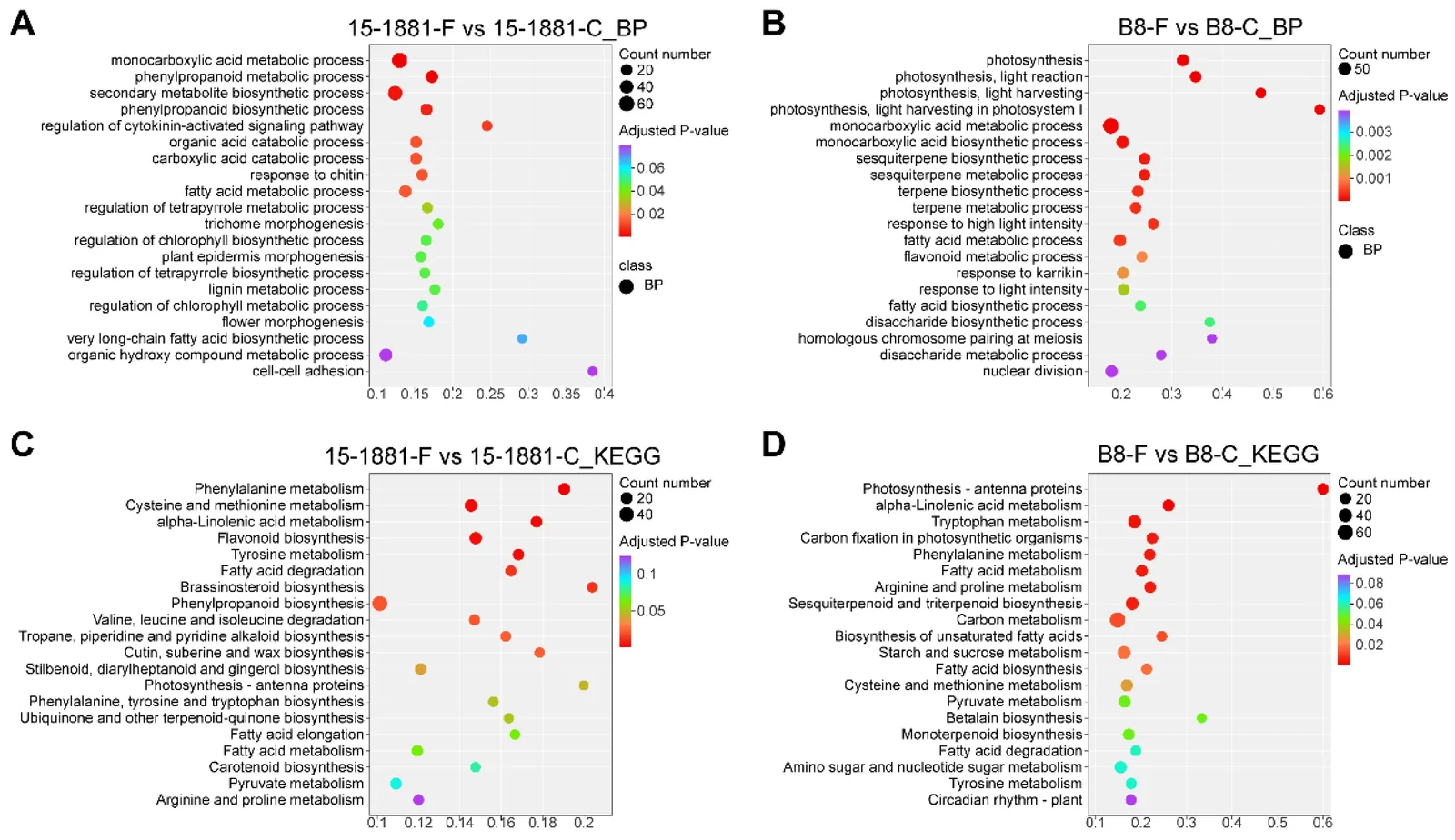
**Gene Ontology (GO) and Kyoto Encyclopedia of Genes and Genomes (KEGG) functional enrichment analyses of DEGs from potato transcriptome.** (**A**) GO enrichment dot plot for DEGs derived from the comparison between 15-1881-F and 15-1881-C. (**B**) GO enrichment dot plot for DEGs derived from the comparison between B8-F and B8-C. (**C**) KEGG enrichment dot plot for DEGs derived from the comparison between 15-1881-F and 15-1881-C. (**D**) KEGG enrichment dot plot for DEGs derived from the comparison between B8-F and B8-C. F = freezing; C = control.

**Figure 8:**
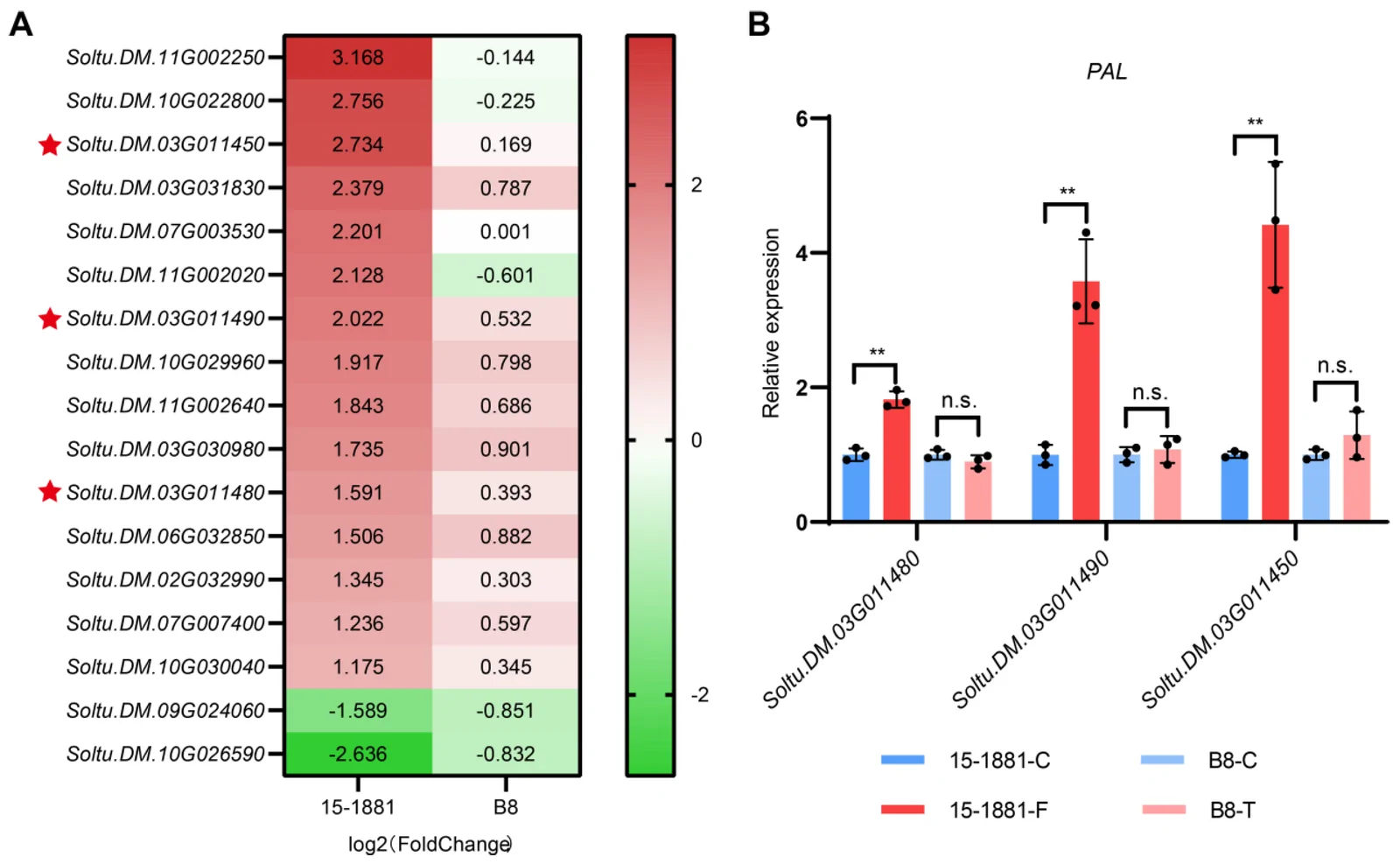
**Candidate genes associated with the freezing response in potato.** (**A**) Fold changes of differentially expressed genes enriched in the phenylpropanoid biosynthesis pathway in accession 15-1881, red asterisks represent phenylalanine ammonia-lyase (*PAL*) genes. (**B**) Relative expression levels of three *PAL* genes in the phenylpropanoid biosynthesis pathway. F = freezing; C = control. *StEF1α* is the internal reference gene; data are presented as means ± SD, *n* = 3; significant differences were determined by Student’s *t*-test, ** denotes statistically significant difference (*p* < 0.01) and n.s. indicates no significant difference.

GO enrichment analysis revealed significant enrichment of monocarboxylic acid metabolism, phenylpropanoid metabolism, and secondary metabolite biosynthetic processes among DEGs from 15-1881 (Figure 7A). Photosynthesis-related pathways dominated the top enriched terms among B8 DEGs (Figure 7B). KEGG enrichment further indicated that phenylpropanoid biosynthesis was the most significantly enriched pathway preferentially altered in 15-1881 under freezing stress (Figure 7C,D). Mapping DEGs onto the phenylpropanoid KEGG pathway revealed that 17 key enzyme genes were significantly upregulated exclusively in 15-1881, with negligible expression changes of their homologs in B8, confirming the specific activation of this pathway in freezing-tolerant potato accession 15-1881 (Figure 8A, Table S5).

As the rate-limiting enzyme of the phenylpropanoid pathway, PAL catalyzes the conversion of L-phenylalanine to *trans*-cinnamic acid and promotes the production of lignin, flavonoid antioxidants, and salicylic acid. We therefore selected three *PAL* genes involved in phenylpropanoid biosynthesis from these 17 genotype-specific (Table S6, Figures S2–S4) DEGs for qRT-PCR validation. The qRT-PCR results show that *Soltu.DM.03G011450*, *Soltu.DM.03G011480*, and *Soltu.DM.03G011490* were significantly upregulated following freezing treatment in 15-1881, whereas no significant upregulation was observed in B8 (Figure 8B). Their expression patterns were highly consistent with the RNA-seq data, supporting the reliability of the transcriptome dataset. These three tandemly arranged genes are promising candidate genes transcriptionally associated with the freezing response in potato.

## 4. Discussion

### 4.1. Application Potential of the FDI Rapid-Screening Workflow and Evaluated Potato Germplasm Resources

In this study, we established a laboratory-based rapid freezing tolerance evaluation system using FDI. This approach alleviates the poor repeatability of field frost phenotyping and reduces the high labor input associated with conventional LT_50_ measurement [10,11,12]. Using this pipeline, five HT accessions were identified as elite parental resources for freezing-tolerant potato breeding. Significant negative correlations between FDI and growth indicators have also been documented in other crops [31,32]. Collectively, these findings indicate that FDI can serve as an effective quantitative index for evaluating crop freezing tolerance. However, this artificial freezing regime differs substantially from natural freezing events under field conditions, which usually feature gradual temperature decreases, variable freezing durations, and temperature fluctuations. Accordingly, the FDI-based phenotyping pipeline developed herein is suitable for rapid preliminary screening to identify potato germplasm with potential freezing tolerance under laboratory conditions. Further field validation under natural freezing environments is required to verify the freezing tolerance and agronomic performance of these candidate accessions.

### 4.2. Divergent Physiological Homeostasis Determines Differential Freezing Tolerance Among Potato Germplasm Accessions

Physiologically, susceptible potato germplasm accessions suffered severe oxidative damage accompanied by excessive ROS and MDA accumulation, whereas tolerant genotypes maintained relatively lower oxidative stress levels, implying potential advantages in ROS homeostasis regulation. Tolerant potato accessions also accumulated higher concentrations of soluble sugar and proline to sustain osmotic homeostasis and limit freezing-triggered electrolyte leakage, consistent with findings in other crops [30]. In this study, chlorophyll a declined more markedly than chlorophyll b in freezing-susceptible accessions under short-term freezing stress. However, because systematic photosynthetic physiological indices were not quantified in the present study, the causal relationship between chlorophyll decline and photosynthetic impairment remains to be further verified. Collectively, the distinct morphological and physiological variations observed between contrasting genotypes provide a phenotypic foundation for screening freezing-tolerant germplasm and exploring associated candidate genes.

### 4.3. Comparative Transcriptomics Reveals Phenylpropanoid Biosynthesis Associated with Freezing Responses in Potato

Comparative transcriptome profiling between contrasting HT and HS genotypes indicated that phenylpropanoid biosynthesis was significantly enriched among genotype-specific DEGs identified in the freezing-tolerant accession 15-1881. This pathway contributes to the biosynthesis of flavonoids, lignin, and phenolic secondary metabolites capable of scavenging excess ROS and stabilizing cell membrane structure, which is consistent with previous freezing stress studies in tomato and other Solanaceae species [33,34]. PAL encodes the rate-limiting enzyme of the phenylpropanoid pathway [35,36]. The obvious transcriptional upregulation of three *PAL* genes in the freezing-tolerant genotype 15-1881 indicates that these genes are associated with potato freezing responses. In contrast, *PAL* genes exhibited negligible expression changes in the freezing-susceptible line B8.

Notably, the transcriptomic comparison in this study was performed using only one freezing-tolerant and one freezing-susceptible genotype. Consequently, the observed differential gene expression patterns may be partially confounded by intrinsic genetic background variations that are independent of freezing stress responses. Moreover, the RNA-seq experiment used a milder freezing treatment (−1 °C) than the phenotypic screening (−4 °C), which may capture different stress response phases. Therefore, the candidate genes identified here should be validated in a larger panel of contrasting genotypes and under conditions matching the phenotypic screening protocol.

## 5. Conclusions

In this study, we detected substantial variation in freezing tolerance among 73 potato germplasm accessions and successfully identified 5 highly freezing-tolerant and 12 highly susceptible accessions. Transcriptomic comparison revealed that the freezing-tolerant potato line 15-1881 exhibited more DEGs under freezing stress. The phenylpropanoid biosynthesis pathway, together with three tandem *PAL* genes, represents a candidate regulatory module potentially associated with freezing tolerance in potato. These findings provide valuable germplasm resources and candidate genes for future freezing-tolerance research and molecular breeding in potato and offer preliminary insights into the transcriptional basis of potato freezing stress responses.

## Figures and Tables

**Table 1:** Freezing tolerance of all tested potato germplasm accessions.

Name/ID	FDI	Freezing Tolerance	Name/ID	FDI	Freezing Tolerance
E2-3	0.19	HT	B2417a	0.52	MT
Huainingxiaotudou	0.19	HT	FJS	0.56	MT
15-1881	0.19	HT	18-6-16	0.56	MT
16HF001	0.19	HT	Kexin18	0.56	MT
Youjin	0.19	HT	Zhongshu1	0.56	MT
E1-3	0.22	T	B18	0.59	MT
G2	0.22	T	Benshu11	0.59	MT
WJ2	0.22	T	Zhengshu6	0.59	MT
Xuechuanhong	0.22	T	Zhongshuzao45	0.59	MT
Zhongshu143	0.22	T	G4	0.63	S
Zhongshu5	0.22	T	G9	0.63	S
N191	0.26	T	Zaodabai	0.63	S
2020341022	0.30	T	Zhongshu20	0.63	S
15-773	0.30	T	Zhongshu3	0.63	S
Atlantic	0.30	T	R1	0.67	S
Zhongshu209	0.30	T	08HE042	0.67	S
B6	0.33	T	N190	0.67	S
E3-4	0.33	T	B3	0.70	S
SDII	0.33	T	B5	0.70	S
15-1794	0.33	T	Longshu7	0.70	S
2-51	0.33	T	B13	0.74	S
Jizhangshu12	0.33	T	458-9	0.74	S
Longshu4	0.33	T	Hongmei	0.74	S
G1	0.37	T	E4-1	0.78	S
Hecuntudou	0.37	T	A1	0.81	HS
1-4	0.37	T	B21	0.81	HS
07HF167	0.37	T	NFJ6	0.81	HS
Hongxishuai	0.37	T	2-9	0.81	HS
1-2	0.41	MT	Favorita	0.81	HS
2019344036	0.41	MT	Hecunzitudou	0.85	HS
Wotu5	0.41	MT	B2414a	0.85	HS
DP	0.44	MT	P340828001	0.89	HS
B2	0.48	MT	WJ1	0.89	HS
B248a	0.50	MT	2-46	0.96	HS
CHAO	0.52	MT	B8	1.00	HS
R2	0.52	MT	E4-5	1.00	HS
24-552	0.52	MT			

## Data Availability

The original contributions presented in this study are included in the article/Supplementary Materials. Further inquiries can be directed to the corresponding authors.

## Supplementary Materials

The following supporting information can be downloaded at: https://www.mdpi.com/article/10.3390/biom16091249/s1, Figure S1: Numbers of potato germplasm accessions with different freezing tolerance levels across various resource types; Figure S2: Chromosome locations and gene structures of three *PAL* genes; Figure S3: Conserved motifs of three phenylalanine ammonia-lyase; Figure S4: Phylogenetic analysis of three phenylalanine ammonia-lyase; Table S1: Basic information of all tested potato germplasm accessions; Table S2: Classification standard of freezing injury grade for potato seedlings; Table S3: Evaluation standard for freezing tolerance of potato seedlings; Table S4: Transcriptome quality control; Table S5: Transcriptomic expression levels and basic information of 17 genes related to phenylpropanoid biosynthesis; Table S6: Basic information of three *PAL* genes; Table S7: Primers used for quantitative real-time PCR analysis in this study.
