# Supplementary material for: Germplasm Screening and Transcriptome Profiling Identify Phenylpropanoid Biosynthesis-Related PAL Candidate Genes Associated with Freezing Tolerance in Potato
Source: Biomolecules. 2026 Aug 28;16(9):1249. doi: 10.3390/biom16091249 (PMC13604389; doi:10.3390/biom16091249)
Supplement: Supplementary file 1 [file biomolecules-16-01249-s001.zip › biomolecules-4484796-supplementary.pdf]

Supplementary Materials

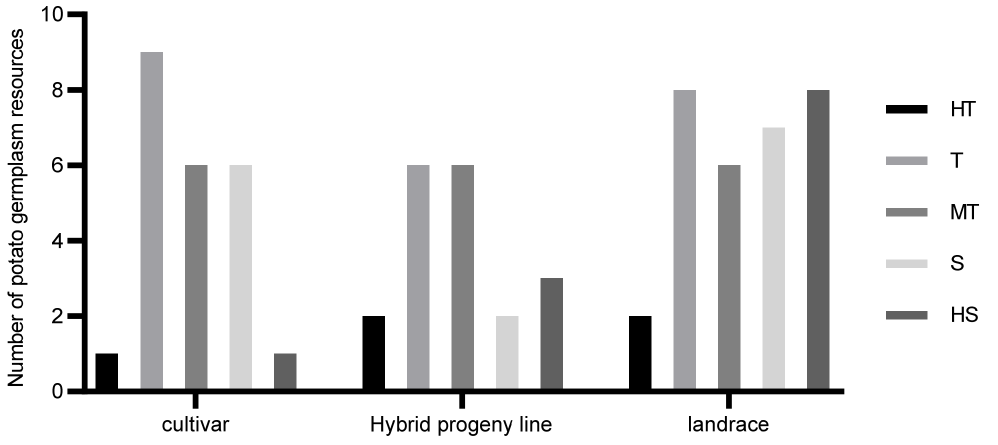

Figure S1　Numbers of potato germplasm accessions with different freezing tolerance levels across various resource types

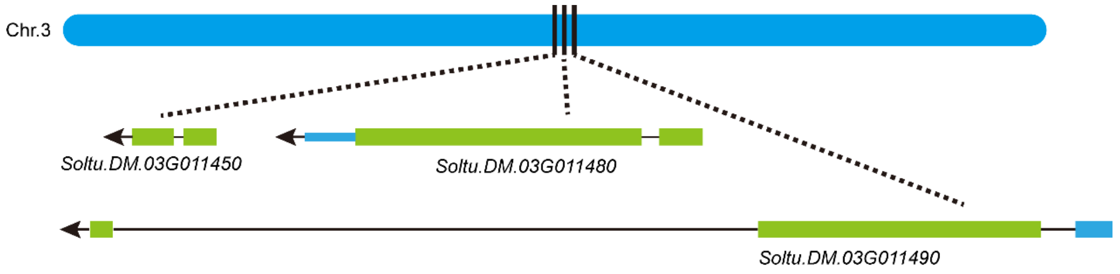

Figure S2　Chromosome locations and gene structures of three *PAL* genes

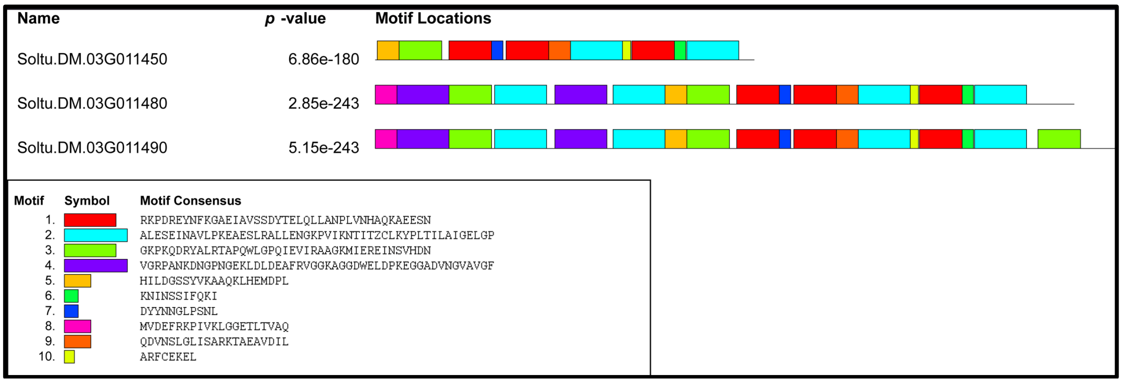

Figure S3　Conserved motifs of three phenylalanine ammonia-lyase

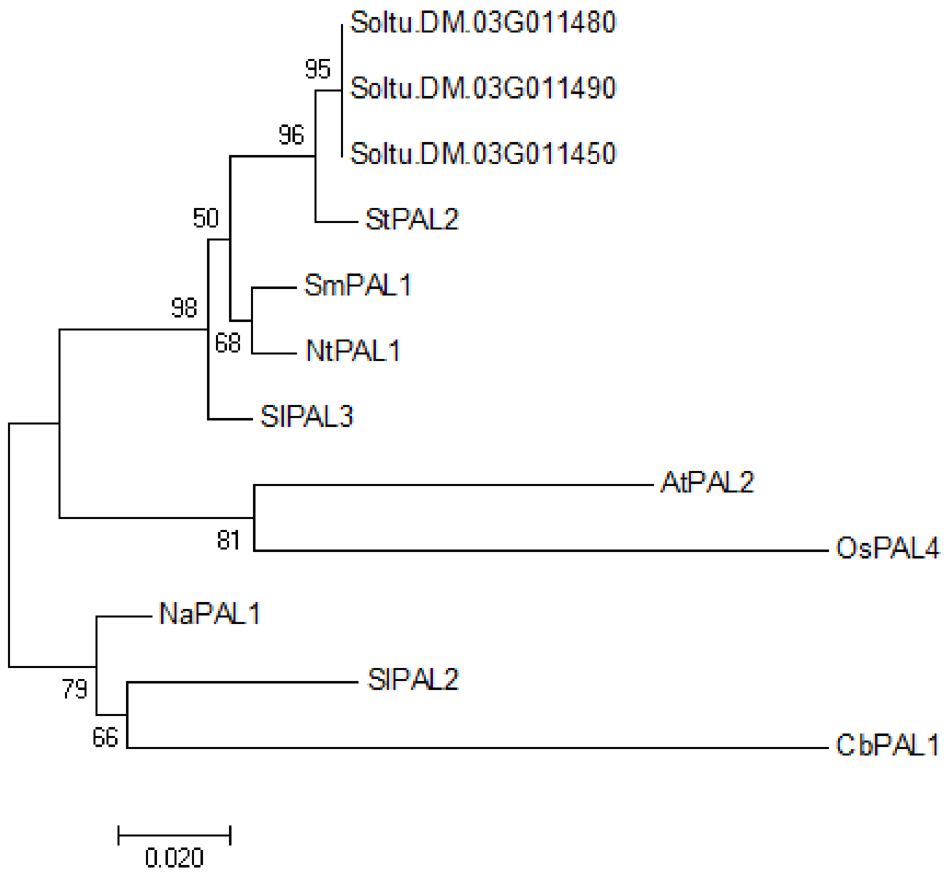

Figure S4 Phylogenetic analysis of three phenylalanine ammonia-lyase

Table S1 Basic information of all tested potato germplasm accessions

No.	Name/ID	Category	No.	Name/ID	Category
1	G1	landrace	38	Zhongshu3	cultivar
2	G2	landrace	39	Zhongshu5	cultivar
3	G4	landrace	40	Zhongshu20	cultivar
4	G9	landrace	41	Zhongshu143	cultivar
5	DP	landrace	42	Zhongshu209	cultivar
6	FJS	landrace	43	Zhongshuzao45	cultivar
7	NFJ6	landrace	44	Zhengshu6	cultivar
8	SDII	landrace	45	Longshu4	cultivar
9	P340828001	landrace	46	Longshu7	cultivar
10	Hecuntudou	landrace	47	Wotu5	cultivar

11	Hecunzitudou	landrace	48	Benshu11	cultivar
12	Huainingxiaotudou	landrace	49	Kexing18	cultivar
13	A1	landrace	50	Jizhangshu12	cultivar
14	B2	landrace	51	Favorita	cultivar
15	B3	landrace	52	Atlantic	cultivar
16	B5	landrace	53	Hongxishuai	cultivar
17	B6	landrace	54	Xuechuanhong	cultivar
18	B8	landrace	55	1-2	Hybrid progeny line
19	B13	landrace	56	1-4	Hybrid progeny line
20	B18	landrace	57	2-9	Hybrid progeny line
21	B21	landrace	58	2-46	Hybrid progeny line
22	R1	landrace	59	2-51	Hybrid progeny line
23	R2	landrace	60	15-773	Hybrid progeny line
24	WJ1	landrace	61	15-1794	Hybrid progeny line
25	WJ2	landrace	62	15-1881	Hybrid progeny line
26	CHAO	landrace	63	24-552	Hybrid progeny line
27	E1-3	landrace	64	458-9	Hybrid progeny line
28	E2-3	landrace	65	18-6-16	Hybrid progeny line
29	E3-4	landrace	66	07HF167	Hybrid progeny line
30	E4-1	landrace	67	08HE042	Hybrid progeny line
31	E4-5	landrace	68	16HF001	Hybrid progeny line
32	Hongmei	cultivar	69	2019344036	Hybrid progeny line
33	Youjin	cultivar	70	2020341022	Hybrid progeny line
34	Zaodabi	cultivar	71	B248a	Hybrid progeny line
35	N190	cultivar	72	B2414a	Hybrid progeny line
36	N191	cultivar	73	B2417a	Hybrid progeny line
37	Zhongshu1	cultivar			

Table S2 Classification standard of freezing injury grade for potato seedlings

Freezing injury grade	Freezing injury symptom
1	No or nearly no freezing damage on leaves and aboveground stems; plants grow normally after recovery
3	Slight freezing injury on the edge of partial leaves; plant growth nearly normal after recovery
5	Partial leaves or upper stems suffer freezing injury; plant growth retarded after recovery
7	Most leaves and upper stems are damaged by freezing; plant growth severely inhibited
9	Plants dead or nearly dead; no new buds regenerated after recovery

Table S3 Evaluation standard for freezing tolerance of potato seedlings

Freezing tolerance grade	Freezing damage index (FDI)	Freezing tolerance
1	$0.00 < \text{FDI} \leq 0.20$	HT
3	$0.20 < \text{FDI} \leq 0.40$	T
5	$0.40 < \text{FDI} \leq 0.60$	MT
7	$0.60 < \text{FDI} \leq 0.80$	S
9	$0.80 < \text{FDI} \leq 1.00$	HS

Highly freezing-tolerant (HT), freezing-tolerant (T), moderately freezing-tolerant (MT), susceptible (S), highly susceptible (HS).

Table S4 Transcriptome quality control

Sample	raw_reads	clean_reads	raw_data(G)	clean_data(G)	error_rate(%)	Q20(%)	Q30(%)	GC_content(%)
15-1881_C_S1	26837806	26837272	8.05	8.01	0.02	97.99	94.75	35.52
15-1881_C_S2	23603345	23602806	7.08	7.05	0.02	98.28	95.38	35.02
15-1881_C_S3	28173508	28172732	8.45	8.42	0.02	98.12	95.02	36.9
15-1881_F_S1	24414973	24414408	7.32	7.27	0.02	97.86	94.4	38.4
15-1881_F_S2	19851425	19850934	5.96	5.93	0.02	98.06	94.86	37.2
15-1881_F_S3	22311795	22311129	6.69	6.67	0.02	97.85	94.29	39.14
B8_C_S1	24160120	24159584	7.25	7.22	0.02	97.95	94.58	36.93
B8_C_S2	25058132	25057516	7.52	7.48	0.02	98	94.71	36.68
B8_C_S3	22861889	22861383	6.86	6.83	0.02	97.97	94.65	36.43
B8_F_S1	21441136	21440442	6.43	6.39	0.02	98.03	94.8	39.31
B8_F_S2	25740600	25739923	7.72	7.68	0.02	98.09	94.92	37.95
B8_F_S3	23224657	23224071	6.97	6.94	0.02	98.05	94.84	36.58

Table S5 Transcriptomic expression levels and basic information of 17 genes related to phenylpropanoid biosynthesis

**Gene ID**	**15-1881_F_S**	**15-1881_C_S**	**log2FC**	***padj***	**B8_F_S**	**B8_C_S**	**log2FC**	***padj***	**Annotation**
Soltu.DM.02G032990	58.55	23.06	1.33	2.46E-03	42.30	34.29	0.30	5.71E-01	NAD(P)H-binding
Soltu.DM.03G011450	106.66	16.03	2.72	1.23E-13	37.14	33.04	0.17	7.73E-01	Phenylalanine ammonia-lyase
Soltu.DM.03G011480	344.04	114.22	1.59	2.12E-11	127.08	96.79	0.39	1.89E-01	Phenylalanine ammonia-lyase
Soltu.DM.03G011490	271.05	66.75	2.03	1.38E-10	78.39	54.21	0.54	1.68E-01	Phenylalanine ammonia-lyase
Soltu.DM.03G030980	279.89	84.07	1.73	5.77E-18	241.73	129.48	0.90	1.40E-04	Cinnamoyl-CoA reductase
Soltu.DM.03G031830	1345.72	258.62	2.38	1.59E-111	782.03	453.12	0.79	3.01E-11	shikimate
Soltu.DM.06G032850	2250.29	792.15	1.51	2.64E-58	1977.28	1072.76	0.88	5.87E-26	Belongs to the cytochrome P450 family
Soltu.DM.07G003530	36.52	7.95	2.17	1.32E-03	21.25	21.23	0.00	9.98E-01	Plastocyanin-like domain
Soltu.DM.07G007400	3505.37	1487.72	1.24	2.36E-56	2579.00	1705.55	0.60	2.35E-16	belongs to the flavoprotein pyridine nucleotide cytochrome reductase family
Soltu.DM.09G024060	6.10	18.33	-1.60	4.53E-02	15.15	27.32	-0.85	1.69E-01	Carboxylesterase
Soltu.DM.10G022800	26.93	3.99	2.79	2.67E-03	21.41	25.03	-0.22	7.84E-01	Lignin degradation and detoxification of lignin-derived products
Soltu.DM.10G026590	443.58	2757.41	-2.64	8.36E-157	1041.03	1853.66	-0.83	1.44E-23	cytochrome P450
Soltu.DM.10G029960	385.14	101.96	1.92	3.28E-17	440.32	253.19	0.80	8.80E-09	Belongs to the cytochrome P450 family
Soltu.DM.10G030040	64.37	28.51	1.18	3.38E-03	69.87	55.02	0.34	4.16E-01	P-coumaroyl quinate shikimate 3'-hydroxylase
Soltu.DM.11G002020	104.19	23.84	2.13	1.28E-10	49.70	75.37	-0.60	9.02E-02	Zinc-binding dehydrogenase
Soltu.DM.11G002250	1256.58	139.82	3.16	6.94E-123	85.10	94.01	-0.15	6.92E-01	Zinc-binding dehydrogenase
Soltu.DM.11G002640	266.31	74.25	1.85	6.56E-17	267.15	166.10	0.69	1.91E-04	Alcohol dehydrogenase GroES-like domain

Table S6　Basic information of three PAL genes

Gene ID	Chr.	Start	End	CDS_length	Protein length	Theoretical pI	Molecular weight	annotation
Soltu.DM.03G011450	chr03	31838629	31839726	1098	365	5.97	40578.15	Phenylalanine ammonia-lyase
Soltu.DM.03G011480	chr03	31874688	31877150	2022	673	6.21	73124.54	Phenylalanine ammonia-lyase
Soltu.DM.03G011490	chr03	31895849	31902002	2124	713	7.97	77929.94	Phenylalanine ammonia-lyase

Table S7 Primers used for quantitative real-time PCR analysis in this study

**Primer name**	**Sequence (5′→3′)**
qStEF1α-F	ATTGGAAATGGATATGCACCA
qStEF1α-R	TCCTTACCTGAACGCCTGTCA
qSoltu.DM.03G011450-F	CCTAAAGAAGTTGAGAGTGC
qSoltu.DM.03G011450-R	TCAATCTCCCTCGCCCG
qSoltu.DM.03G011480-F	AAAAGTTCGATCGCCGGG
qSoltu.DM.03G011480-R	TAGCAGATTGGAAGAGGAGC
qSoltu.DM.03G011490-F	GAGCTTCGTTTTTCAGATCC
qSoltu.DM.03G011490-R	TCAAGTTGGAGGGGGAAGC
